# Trends in mental health referrals before, during and after the COVID-19 pandemic: retrospective population-based service evaluation in a single NHS trust

**DOI:** 10.1192/bjo.2025.10972

**Published:** 2026-02-09

**Authors:** Zahir Zubair Shah, Shanquan Chen, Tamsin J. Ford, Catherine M. Walsh, Jonathon Artingstall, Benjamin R. Underwood

**Affiliations:** School of Clinical Medicine, University of Cambridge, Cambridge, UK; School of Public Health, LKS Faculty of Medicine, The University of Hong Kong, Hong Kong, China; Department of Psychiatry, University of Cambridge, Cambridge, UK; Cambridgeshire and Peterborough NHS Foundation Trust, Windsor Unit, Fulbourn Hospital, Cambridge, UK

**Keywords:** Mental health services, old age psychiatry, general adult psychiatry, child and adolescent psychiatry, COVID-19

## Abstract

**Background:**

The COVID-19 pandemic caused by the SARS-CoV-2 virus was the biggest global health emergency in the past century. The impact of the pandemic on society, and demand for health services, especially mental health services is not fully understood.

**Aims:**

We describe the change in activity in a single UK NHS mental health trust during and following the pandemic.

**Method:**

We conducted a retrospective service evaluation using a population-based referral rates and clinical activity for mental health disorders in Cambridgeshire over the period 1 January 2017 to 31 December 2023. We divided the time period into pre-pandemic period, during the pandemic, and post-pandemic months. A negative binomial regression model was fitted to the monthly rates to yield incidence rate ratios. Subgroup analyses were performed by age, gender, ethnicity, and level of deprivation.

**Results:**

There was a steep decline in both referrals and clinical contacts during the lockdowns with a subsequent and steep increase in these measures during the immediate post-pandemic period. Increased numbers of referrals and contacts have been sustained well into the post-pandemic period.

**Conclusions:**

In a single county-wide mental health service in the UK, the prolonged and sustained increase in both referrals and activity post-pandemic was not matched by equal increase in resource to meet demand. Our findings may be useful to effectively plan mental health services before, during and after any future pandemics.

The COVID-19 pandemic, caused by the SARS CoV-2 virus, was the first global respiratory pandemic since the influenza pandemic of 1918. It was declared a pandemic by the World Health Organization on 11 March 2020 and is estimated to have caused more than 7 million deaths worldwide.^1^ The impact of the pandemic on mental health was the subject of much speculation on social media and in the lay and scientific press.^2^ SARS CoV-2 is neurotropic, and an association has been reported between infection and increased subsequent psychiatric diagnosis.^3^ The pandemic may also have had indirect effects on mental health through lockdowns, loss of social contact, changes in economic circumstances, fear of infection and altered access to healthcare.

One way of examining the totality of these factors is to examine rates of referral to secondary mental healthcare services. National data for England suggest a 25% increase in the number of people in contact with mental health services by 2023 compared with before the pandemic.^4^ National data, however, do not describe local nuance, and studies based on these data are thus limited in terms of detail and interpretation. We previously described changes in mental health service use in Cambridgeshire during lockdowns and in the early stages of the implementation of vaccination.^5,6^ Cambridgeshire is a single county in England covered by a single mental health provider (Cambridgeshire and Peterborough NHS Foundation Trust). The trust provides secondary mental healthcare to a population of approximately 1 million people. Accurate information with respect to changes in the demand for mental health services and the activity of these services is essential for planning future pandemic responses, not just in the acute stage but also post-pandemic. Here, we extend our previous work and describe the changes in referral and activity patterns in one National Health Service (NHS) mental health trust before, during and 2 years after the pandemic.

## Method

### Study design and participants

We conducted a retrospective service evaluation examining population-based referral rates and clinical activity for mental health disorders in Cambridgeshire, spanning the period from 1 January 2017 to 31 December 2023. The data did not describe individual patents, had already been collected by the trust, and was available for the purpose of audit and evaluation. It could be made available to the public if requested through a freedom of information request and only describes what had happened in our specific service. As such, the data were extracted as part of an internally trust-approved service evaluation rather than using the rubric of research. We identified the following key time periods related to national lockdowns: first (23 March 2020 to 10 May 2020), second (5 November 2020 to 2 December 2020) and third (4 January 2021 to 29 March 2021). Restrictions were gradually lifted after the third lockdown and fully removed from 1 April 2022. During the lockdown periods, non-essential businesses were closed, and stay-at-home orders were enforced.

### Data examined

The primary outcome was the number of monthly referrals for mental disorders, enumerated for each month from January 2017 to December 2023. The referral data were broken down by age (0–19 years: children and adolescents; 20–44 years: working-age young adults; 45–64 years: working-age older adults; and ≥65 years), gender, ethnicity (White, ethnic minorities or unknown) and Index of Multiple Deprivation.

Other outcomes included contacts with mental health services. A contact was counted as any point of contact with mental health services, be that face-to-face or online consultations. Weekly contacts were compared across the years pre-pandemic and post-pandemic. These contacts consisted primarily of community-based data but also included some in-patient data. Trust staffing levels were obtained for the time period between July 2018 and the end of 2023 to provide a measure of changing resource against changing demand.

### Statistical analysis

Monthly referral frequencies are reported as means with standard deviations. Monthly rates were calculated per 10 000 using population denominators registered in the Cambridgeshire and Peterborough NHS Foundation Trust catchment area. We divided time into the pre-pandemic period (January 2017–February 2020), which served as the baseline, and the during or post-pandemic months (March 2020 and after), for which each month was compared individually with the baseline pre-pandemic period.

Our analysis sought to identify whether changes might be a result of normal variation. A negative binomial regression model, accounting for overdispersion, was fitted to the monthly rates. Year and month were controlled as continuous variables to account for underlying temporal trends, and each month during the pandemic or post-pandemic periods was included as a categorical variable. This model yielded incidence rate ratios with 95% confidence intervals to determine the statistical significance of temporal changes in monthly referral rates from March 2020 onwards compared with the baseline. To facilitate interpretation of the fitted results, a linear approximation to the negative binomial model was performed by estimating marginal effects and 95% confidence intervals for the means, representing the changes in monthly referrals per 10 000 using population compared with the same month in the pre-COVID-19 period. The Breusch–Godfrey and Breusch–Pagan tests were used to assess autocorrelation and heteroskedasticity of residuals, respectively. Subgroup analyses were conducted by age, gender, ethnicity and level of deprivation to understand changes in service use in these sub-populations. *P*-values for between-group comparisons were derived from negative binomial regression models, with the corresponding counts in each period serving as the outcome variable and the period (with pre-COVID-19 as the reference) as the primary predictor variable. All analyses were performed using R version 4.3.0 for macOS (The R Foundation, Vienna, Austria; https://cran.r-project.org/bin/macosx/).

## Results

### Overall changes in referral numbers

**Fig. 1:**
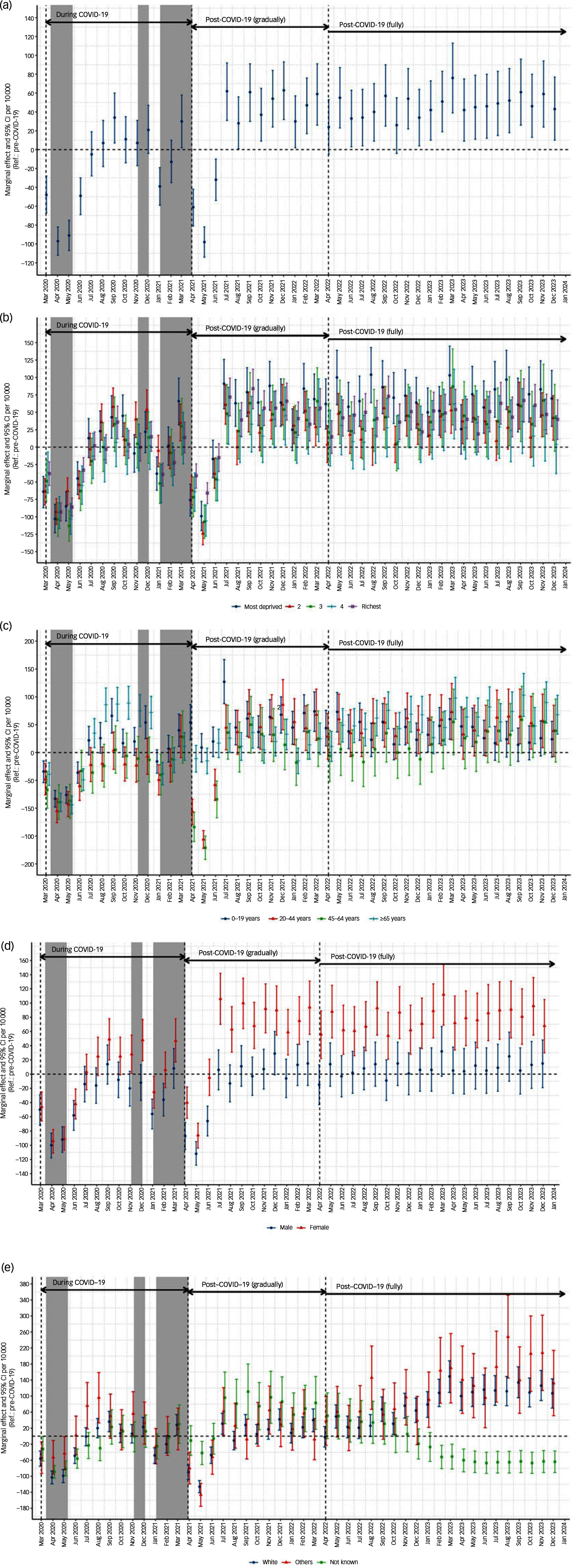
Changes in numbers of referrals. Numbers are standardised by rate per 10 000 population, and 95% confidence intervals are shown, with the reference set at pre-pandemic levels, for (a) total referrals to the trust, (b) referrals by quintile of deprivation, (c) referrals by age group, (d) referrals by gender and (e) referrals by ethnicity. Note the significant and sustained increase in referrals post-pandemic, most strikingly in women.

The changes in overall numbers of referrals in total and by age, gender and level of deprivation, relative to pre-pandemic levels, are shown in Fig. 1 and Table 1. Total referrals showed a dramatic decline during lockdown but then quickly returned to well above pre-pandemic levels and remained high relative to pre-pandemic rates for nearly 2 years post-pandemic. Although the greatest increase in referrals immediately post-lockdown was at the extremes of age, the sustained post pandemic increase was seen more widely across all age groups and levels of deprivation. These changes were greatest among women, those from areas with the highest levels of deprivation, and those of Black or minority ethnic groups (Fig. 1).

**Table 1:** Statistical comparison of referrals broken down by age group and contacts pre, during and post-pandemic during vaccination (April 2021–April 2022) and post vaccination (after April 2022)^a^

Variable	Pre-COVID-19 Mean (s.d.)	During COVID-19 Mean (s.d.), *P* ^b^	Post-COVID-19 emerging from pandemic April 2021 to April 2022 Mean (s.d.), *P* ^b^	Post-COVID-19 post-pandemic (full vaccination) April 2022–2024 Mean (s.d.), *P* ^b^
Total referrals	5619.8 (337.1)	5925.5 (1218.4), 0.16	8623.1 (704.3), <0.001	9675.3 (835.9), <0.001
Total contacts	20890.5 (1660.5)	19206.4 (2011), 0.002	25385.3 (2107.4), <0.001	28028.5 (1883.9), <0.001
Referrals for 0–19	555.8 (135.2)	817.2 (221.6), <0.001	1184.2 (228.7), <0.001	1513.4 (260.5), <0.001
Referrals for 20–44	2693.9 (172)	2716.8 (549.3), 0.819	4226.2 (357.2), <0.001	4513.6 (376.5), <0.001
Referrals for 45–64	1321 (97.6)	1225.6 (193.1), 0.022	1769.3 (138.8), <0.001	1902.6 (161.9), <0.001
Referrals for ≥65	1049 (66.3)	1165.9 (306.6), 0.02	1443.4 (113.4), <0.001	1745.7 (148.8), <0.001

a. All cells show data per month.

b. *P*-values were derived from negative binomial regression models, with the corresponding counts in each period serving as the outcome variable and the period (with pre-COVID-19 as the reference) as the primary predictor variable.

### Service activity

**Fig. 2:**
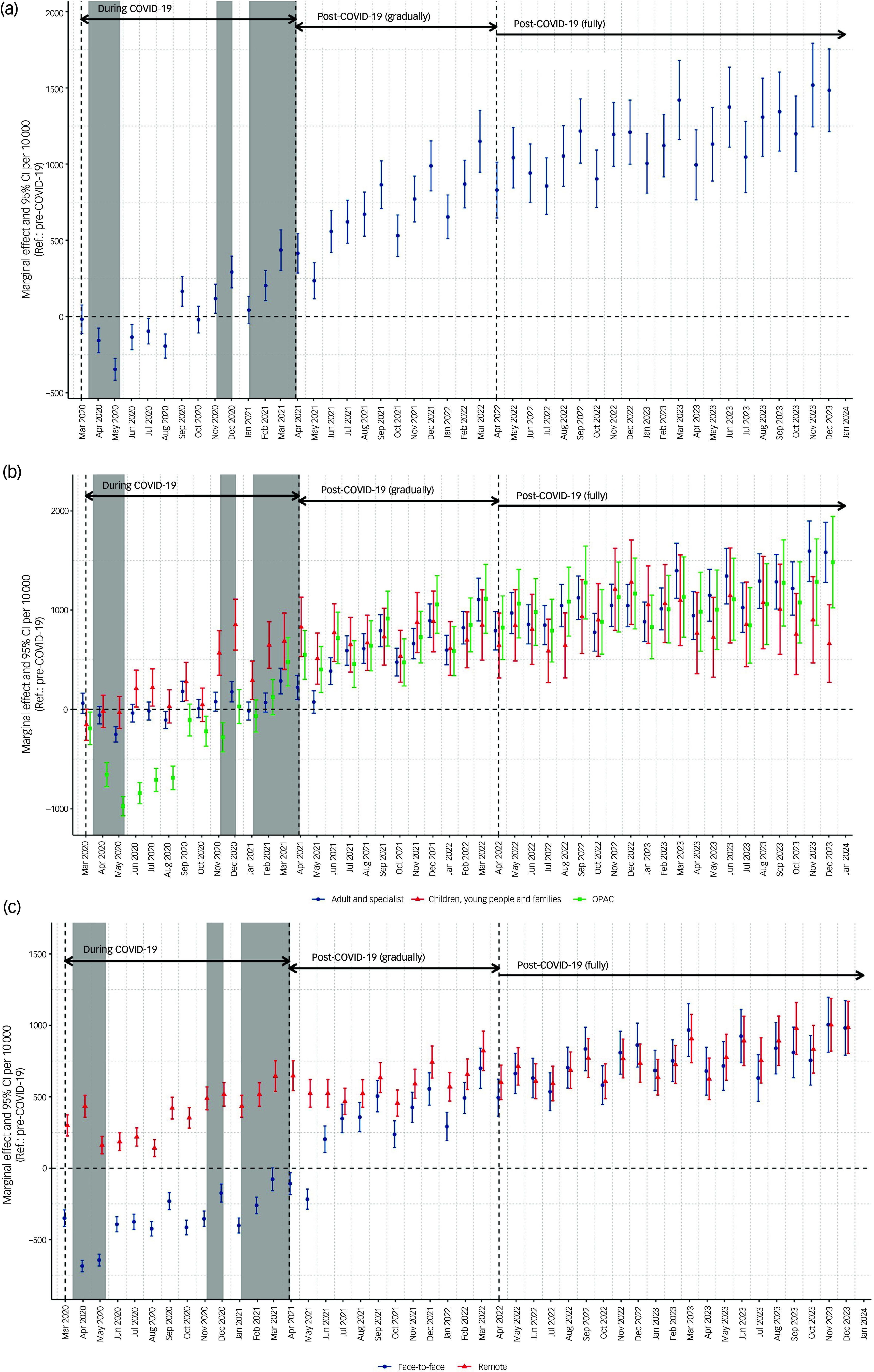
Changes in numbers of contacts. Numbers are standardised by rate per 10 000 population, and 95% confidence intervals are shown, with the reference set at pre-pandemic levels, for (a) total staff contacts, (b) contacts by clinical directorate (based on age: broadly, children and young people aged 0–18 years; adult and specialist services for ages 18–65 years; and older people and adult community >65 years of age), and (c) contacts made face-to-face versus remotely. Note the sustained increase in contacts post-pandemic both face-to-face and remotely and in all age groups, though with a greater increase in face-to-face contacts. OPAC, older people and adult community.

To investigate whether increased rates of referral were matched by increased activity, we examined contacts in mental health services, a measure of activity between clinicians and patients. We found significantly higher contacts post-pandemic, both overall and for each of the three directorates covering the age range. Although the greatest impact on reduced contacts was for older people during lockdown, an increase in contacts was seen at all ages post-lockdown and post-pandemic. To understand the impact of new ways of working on increased service activity, we examined changes in remote versus face-to-face contacts. During lockdown, there was a decrease in face-to-face contacts and an increase in remote contacts compared with pre-pandemic. Both methods of contact increased, but the greatest change was in face-to-face contacts after lockdown and in the post pandemic period. These results are summarised in Fig. 2. Differences in referrals and contacts among the pre-pandemic, during-pandemic and post-pandemic periods were all highly significant and are shown in Table 1.

### Staffing levels

**Fig. 3:**
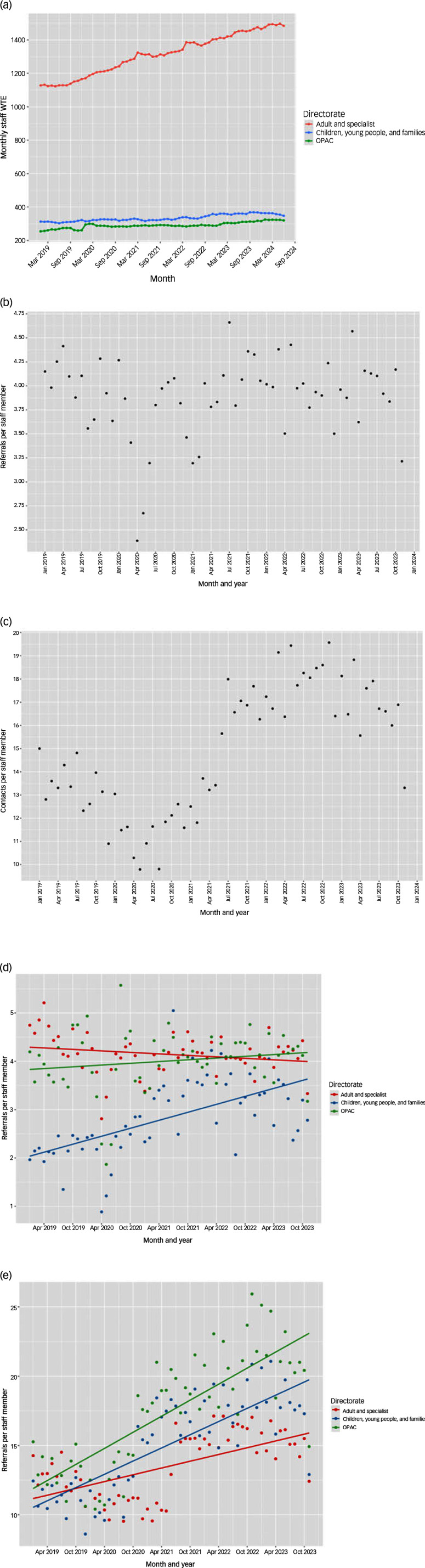
Changes in numbers of referrals and contacts per staff member over time. Results are shown for (a) total staff numbers per directorate over time, (b) referrals and (c) contacts per staff member over time for the whole trust, as well as (d) referrals and (e) contacts per staff member by directorate. WTE, whole time equivalent; OPAC, older people and adult community.

We sought to investigate whether staffing levels had increased to meet this increased activity. There were modest increases in staff numbers overall. We examined monthly contacts in total, and broken down by directorate (children, adults and older people) and number of staff employed in the clinical directorates. These data, which are presented in Fig. 3, showed a sustained increase in contacts per staff member post-pandemic in the clinical teams. We found a sharp decrease in the number of referrals made per staff member at the height of the pandemic, with a return to pre-pandemic levels afterwards. When the results were broken down by directorate, the same effect was seen, except for the children, young people and families group, for which there was a small but sustained increase in number of referrals per staff member post-pandemic.

## Discussion

To our knowledge, this is the most complete description of the impact of the SARS-CoV-2 pandemic on mental health referrals and activity in a single UK NHS trust. We found decreases in numbers of referrals and face-to-face contacts during lockdown, followed by consistently increased rates of referral following the pandemic in all age groups, particularly among women. It is interesting to speculate why this might have been the case: whether it was caused by a direct effect of viral infection on the brain; indirect effects of viral infection (for example, fear of infection and/or death) or interventions to tackle it (for example, decreased social contact due to lockdown); indirectly through economic impact, increased awareness and help-seeking; or a combination of these. The increase in referrals led to a significant increase in activity, as measured by contacts (both remote and face-to-face) per clinical staff member despite smaller increases in staffing, suggesting increased productivity. This may have been partly facilitated by new ways of working that were accelerated by the pandemic, such as remote assessment and treatment. The biggest increase was for face-to-face contacts, which suggests that staff have had to absorb increased workload. Although this indicates that increased productivity is possible and can be sustained, there will be limits to how much productivity can be increased without extra resources. There may also be costs associated with sustained increased productivity, including staff burnout and challenges to retention, particularly in a workforce recovering from the challenges of working through a pandemic.

There was a decrease in numbers of referrals during lockdown, which might represent a change in patient behaviour and service availability, including in primary care. An exception to this was the increase in numbers of referrals for young people during and shortly after lockdown; this finding may suggest a particularly deleterious effect of this intervention on the mental health of young people and their carers, who were often trying to work while supporting remote schooling. These roles tended to fall disproportionately on women, which also might explain the higher rates of referral among women.^7^

Our evaluation had some limitations. Although we have speculated on causes for the trends we observed, our study was retrospective in nature and so could show only association, not causation, and was subject to potential confounding. As our results came from a single site, it is possible that they are not widely applicable to other parts of the UK or beyond, owing to local effects, although the population covered by this single service is large. Cambridgeshire is an area with a predominantly White population, living in urban, semi-rural and rural areas. The full range of deprivation was represented, but we were limited in our ability both to analyse the results by ethnicity and to extrapolate the results to more diverse populations, such as those living in metropolitan cities. The trust moved to a new electronic patient record in December 2020, and it is possible this changed recording of clinician activity, although it seems unlikely that this would have affected recording of referrals.

Despite these caveats, our evaluation provides a description of the impact of a respiratory viral pandemic on mental health referrals and activity before, during and after a pandemic in a single county in England. This work could be of use for planning services in future pandemics, particularly for services in Cambridgeshire. For example, outside the mental health system, the use of ‘trusted’ adults to provide schooling and/or childcare for at least part of the week might relieve pressure on vulnerable families.^8^ Within mental health services, consideration might be given to moving resource to young people’s services during periods of lockdown, and future pandemic responses should include increased provision for mental health services post-pandemic. Our data are also helpful for organising and resourcing mental health services now. We describe a sustained increase in in demand for mental health services across the age range. This increased demand has not been matched by a similar increase in staffing, which suggests the existing workforce is doing more than pre-pandemic. To continue to meet the sustained increases in pressure, or to meet any further increases in demand, more resources may be required.

## Data Availability

The data that support the findings of this study are available from the corresponding author, Z.Z.S., upon reasonable request.
